# Piceatannol from Passion Fruit Seed Waste: A Circular Bioeconomy-Driven Pathway Toward a Skin-Targeted Bioactive

**DOI:** 10.3390/ijms27083451

**Published:** 2026-04-12

**Authors:** Dian Zhang, Chuda Chittasupho, Supat Jiranusornkul

**Affiliations:** 1 Department of Immunology, Xi’an Medical University, Xi’an 710021, China; zhangdian@xiyi.edu.cn; 2 Department of Pharmaceutical Sciences, Faculty of Pharmacy, Chiang Mai University, Chiang Mai 50200, Thailand; chuda.c@cmu.ac.th; 3 Clinical Research Center for Food and Herbal Product Trials and Development (CR-FAH), Faculty of Medicine, Chiang Mai University, Chiang Mai 50200,Thailand

**Keywords:** piceatannol, *Passiflora edulis*, seed by-product, agro-waste valorization, circular bioeconomy, cosmeceutical bioactive, skin-healthy ingredient, skin aging

## Abstract

*Passiflora edulis* (passion fruit) seed waste, an abundant by-product of the juice industry, is a promising source of piceatannol (PIC), a hydroxystilbene with superior antioxidant activity compared to resveratrol. However, its translation into a skin-targeted ingredient remains hindered by a lack of standardization and clinical validation. This review synthesizes current evidence on the dermatological potential of PIC and proposes a translational roadmap within a circular bioeconomy framework. Preclinical studies demonstrate that PIC exerts multi-target effects relevant to skin aging and acne, including ROS scavenging, anti-inflammatory activity via NF-κB/MAPK inhibition, suppression of melanogenesis, enhancement of hyaluronic acid and collagen synthesis, and antibacterial action against *Cutibacterium acnes*. However, clinical data are limited and methodologically inconsistent. To bridge this translational gap, we propose a development strategy focused on: (i) extract standardization with a proposed minimum PIC content (e.g., ≥0.3% *w*/*w*); (ii) an integrated biorefinery approach for the co-production of seed oil and phenolic fractions; and (iii) a phase-gate pipeline encompassing dermal safety assessment, advanced delivery optimization, and biomarker-correlated clinical trials.

## 1. Introduction

**Figure 1:**
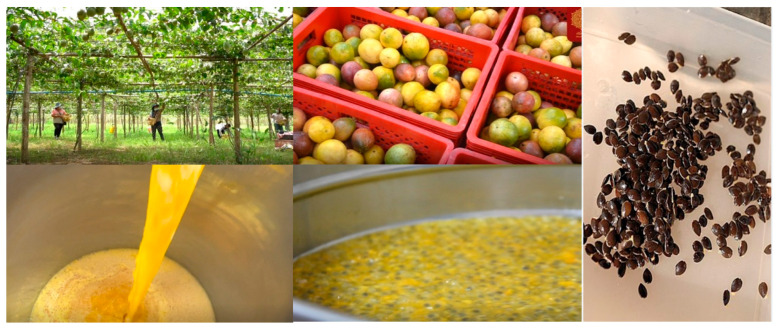
A pictorial representation of passion fruit plantation, juice processing, and seed waste generation. Passion fruits are processed for juice extraction, yielding seeds as a major by-product. These seeds are subsequently used for oil recovery and the extraction of phenolic compounds, including piceatannol, within a circular bioeconomy framework.

The global juice processing industry generates substantial quantities of passion fruit (*Passiflora edulis*) seed waste, an agro-industrial byproduct that is often underutilized or discarded [1,2] (Figure 1). Concurrently, the cosmetic and dermocosmetic sectors are increasingly seeking sustainable, bio-based, and scientifically substantiated active ingredients. Notably, various Passiflora species have already been incorporated into cosmeceutical formulations and patented products due to their abundant phytochemical profiles and skin-relevant bioactivities [3]. This convergence presents a significant opportunity within the circular bioeconomy paradigm, where waste streams can be valorized into high-value products.

**Figure 2:**
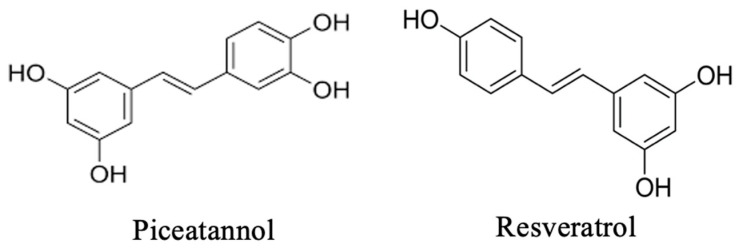
Chemical structures of piceatannol (PIC) and resveratrol. PIC differs from resveratrol by the presence of an additional 3′-hydroxyl group (highlighted), forming a catechol moiety on the B-ring, which confers enhanced antioxidant activity.

Passion fruit seeds (PFSs) have been identified as a potential source of the stilbenoid piceatannol (PIC; trans-3,3′,4,5′-tetrahydroxystilbene), with reported concentrations varying considerably depending on the cultivar and extraction method, reaching levels as high as 3.68% (*w*/*w*) [4,5]. While the biosynthetic origin of PIC in *Passiflora* remains uncharacterized, studies in grapevine (*Vitis vinifera*) demonstrate that PIC can be formed via regiospecific ortho-hydroxylation of resveratrol catalyzed by polyphenol oxidase (PPO) exhibiting cresolase activity, which is a pathway that may operate in other high-PIC-producing plants [6]. Structurally, PIC is a hydroxylated analog of resveratrol and a member of the natural stilbenoid family, distinguished by an additional 3′-hydroxyl group that forms a catechol moiety on its B ring (Figure 2) [7]. This modification confers markedly enhanced antioxidant and anti-inflammatory activities compared to resveratrol, underpinning its multi-target potential in skin health and aging [8,9]. Importantly, this aligns with a growing scientific consensus that plant-derived polyphenols—by modulating conserved longevity pathways such as SIRT/NAD^+^, AMPK, and mTOR—can mitigate age-related functional decline and support healthy aging [10].

Compared with other extensively characterized polyphenols such as resveratrol and quercetin, PIC offers distinct advantages, particularly superior antioxidant capacity attributed to its catechol moiety and a more favorable selectivity for SIRT1 activation. However, these polyphenols also share common limitations, including poor aqueous solubility, rapid phase II metabolism, and photochemical instability. While resveratrol has progressed further in clinical development, its translation has been hindered by poor systemic bioavailability, underscoring the importance of delivery strategies that PIC can leverage from the outset. Notably, the concentration of PIC required for certain in vitro activities remains higher than those typically achieved in vivo, and the current evidence base for PIC in human skin is considerably less developed than that for resveratrol.

Despite promising preclinical data, developing passion fruit seed-derived PIC into a reliable dermocosmetic ingredient faces several translational barriers. These include the lack of standardized extracts with defined critical quality attributes (CQAs), insufficient dermal safety and photostability data, and the absence of clinical trials that correlate efficacy with mechanistic biomarkers. Consequently, no cosmeceutical products currently leverage this waste stream for standardized PIC delivery.

This review is the first to integrate the dermatological science of PIC with a practical, testable development roadmap grounded in circular bioeconomy principles. We aim to: (1) synthesize the evidence for PIC’s multi-target activity in skin aging and acne; (2) critically evaluate existing in vivo and clinical data to identify key evidence gaps; and (3) propose a phase-gate translational framework that prioritizes standardization, sustainable sourcing, nano-delivery, and clinical validation. By doing so, we provide a replicable blueprint for transforming an agro-waste into a scientifically validated, commercially viable skin-targeted bioactive.

## 2. Sources, Chemical Properties, and Delivery Imperatives

PIC (trans-3,3′,4,5′-tetrahydroxystilbene) is a hydroxylated derivative of resveratrol, distinguished by an additional 3′-hydroxyl group that forms a catechol moiety on its B-ring [11,12]. This structure underpins its significantly enhanced antioxidant capacity, with oxygen radical absorbance capacity (ORAC) values several-fold higher than those of resveratrol [13,14]. *Passiflora edulis* (passion fruit) seeds, a major by-product of the global juice industry, constitute an exceptionally rich and sustainable source of PIC [15,16].

The translation of this potential into marketworthy skincare products, however, is challenged by PIC’s inherent physicochemical profile. PIC possesses a molecular weight of 244.24 g/mol and a calculated logP value of approximately 2.5, indicating moderate lipophilicity that theoretically favors skin partitioning. However, its aqueous solubility remains intrinsically low (<0.1 mg/mL), and its tendency to undergo oxidation and photodegradation upon exposure to light and air poses significant challenges for formulation stability and shelf-life [11,17]. Upon oral administration, it undergoes rapid and extensive phase-II metabolism (glucuronidation and sulfation), resulting in low systemic bioavailability and a pharmacokinetic profile that underscores the advantage of topical delivery for direct, localized skin effects [18,19].

To overcome these barriers, advanced delivery systems are required. Encapsulation within nanocarriers such as liposomes, solid lipid nanoparticles (SLNs), nanostructured lipid carriers (NLCs), and polymeric nanoparticles has proven effective in enhancing PIC’s solubility, providing a protective barrier against degradation, and improving its retention and penetration into skin layers [20,21,22,23,24]. Among these nanocarriers, liposomes offer excellent biocompatibility and the ability to encapsulate both hydrophilic and lipophilic compounds, but may suffer from limited stability. SLNs provide enhanced physical stability and controlled release, yet their highly ordered crystalline structure limits drug loading capacity. NLCs, by incorporating liquid lipids, create a less ordered matrix that improves loading capacity and modulates release profiles, making them particularly suitable for PIC. Polymeric nanoparticles, especially those derived from biodegradable polymers such as PLGA or chitosan, offer tunable release kinetics and surface functionalization, but may require more complex manufacturing processes. The choice of delivery system thus depends on the balance between formulation stability, skin penetration enhancement, and commercial scalability. Notably, within an integrated biorefinery concept, the co-produced PFS oil, rich in linoleic acid and tocopherols, could serve as a promising, sustainable, and bioactive lipid phase for formulating PIC-loaded NLCs and is thus considered ideal for such applications [25,26,27]. Recent HS-GC-IMS profiling confirmed that cold-pressed *P. edulis* seed oils contain over 100 volatile aroma compounds—such as esters, alcohols, and ketones (in purple varieties) or aldehydes and pyrazines (in yellow varieties)—imparting desirable fruity, creamy, or nutty notes that may further enhance consumer acceptability in cosmeceutical formulations [28].

## 3. Preclinical Evidence for Multi-Target Dermatological Activity

**Figure 3:**
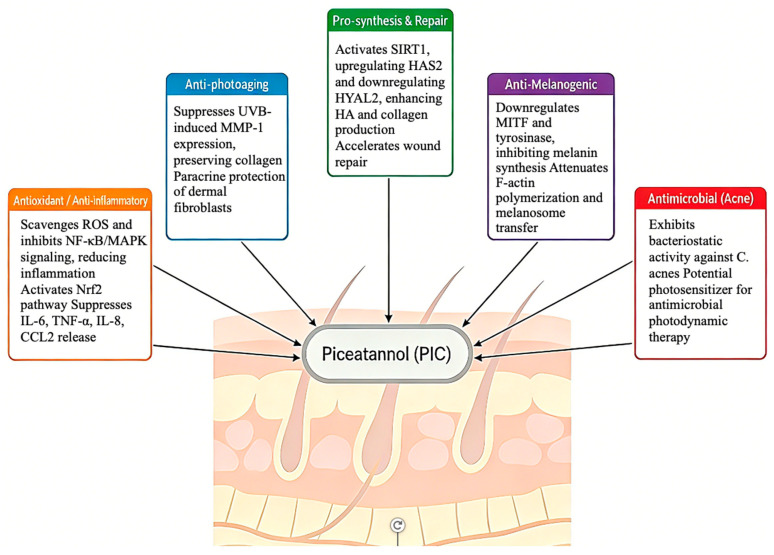
Schematic overview of piceatannol’s multi-target mechanisms in skin biology. PIC modulates five key pathways: (1) Antioxidant/anti-inflammatory: scavenges reactive oxygen species (ROS) and inhibits NF-κB/MAPK signaling; (2) Anti-photoaging: suppresses UVB-induced matrix metalloproteinase-1 (MMP-1) expression, preserving collagen; (3) Pro-synthesis: activates SIRT1, upregulating hyaluronan synthase 2 (HAS2) and promoting hyaluronic acid (HA) and collagen production; (4) Anti-melanogenic: downregulates microphthalmia-associated transcription factor (MITF) and tyrosinase (TYR), inhibiting melanin synthesis; (5) Antimicrobial: exhibits bacteriostatic activity against *Cutibacterium acnes*. This integrated network supports PIC’s potential as a multi-functional cosmeceutical active ingredient.

A robust body of in vitro and ex vivo evidence delineates PIC’s pleiotropic effects across the interconnected pathways driving skin aging and dysfunction, as summarized in Figure 3 and Table 1. Resveratrol and its structural analogues, including PIC, represent a promising class of natural anti-aging agents that target multiple hallmarks of skin aging through shared molecular pathways [29].

### 3.1. Combating Oxidative Stress, Inflammation, and Photoaging

The robust direct free radical scavenging capacity of PIC has been extensively characterized and validated through standardized chemical assay methodologies [30,31,32]. This enhanced antioxidant capacity over resveratrol is structurally attributable to the presence of a catechol (3′,4′-dihydroxy) moiety on its b-ring. Structure–activity relationship studies have demonstrated that trans-resveratrol analogs possessing this 3,4-dihydroxyl configuration exerts markedly greater inhibitory effects on lipid peroxidation compared to resveratrol itself, highlighting the critical role of the ortho-dihydroxy motif in redox stabilization and radical quenching [33].

In biologically relevant skin cell models, the enhanced antioxidant properties of PIC translate into measurable cytoprotective effects. PIC mitigates oxidative stress-induced cellular damage by activating the Nrf2 and SIRT1 pathways [30,31,32]. Concurrently, it exhibits strong anti-inflammatory properties, significantly suppressing the LPS-induced release of pro-inflammatory cytokines (e.g., IL-6, TNF-α, IL-8, CCL2) by inhibiting NF-κB and MAPK signaling pathways in immune and inflammatory cells [33,34,35,36,37]. Notably, piceatannol-rich extracts from *Passiflora edulis* seeds themselves demonstrate significant anti-inflammatory activity, as evidenced by suppression of nitric oxide production in LPS-stimulated macrophages [38].

A key action relevant to skin aging is its protection against UVB-induced photoaging. In UVB-irradiated human keratinocytes, PIC reduces intracellular ROS generation and potently downregulates the expression and activity of matrix metalloproteinase-1 (MMP-1), a major collagen-degrading enzyme implicated in photoaging [39,40,41]. Importantly, a paracrine protective effect has been observed, where conditioned medium from PIC-treated, UVB-exposed keratinocytes loses its capacity to induce MMP-1 in co-cultured dermal fibroblasts, thereby helping to preserve dermal extracellular matrix integrity [23,41].

### 3.2. Promoting Skin Biosynthesis and Repair: A Dual Role

Beyond its cytoprotective properties, PIC actively stimulates the biosynthesis of essential dermal components. In human dermal fibroblasts, PIC upregulates hyaluronan synthase 2 (HAS2) while downregulating hyaluronidase 2 (Hyal2) in a SIRT1-dependent manner, leading to a net increase in hyaluronic acid synthesis—a critical factor for skin hydration and volume [42]. Additionally, PIC-rich PFS extract has been shown to increase total soluble collagen production; this effect is abolished upon polyphenol depletion, thereby confirming PIC’s role as a principal driver of collagen biosynthesis [16,43]. Furthermore, such extracts accelerate scratch-wound closure in keratinocyte monolayers, indicating pro-repair activities [43].

While the in vitro studies summarized above provide mechanistic insights into PIC’s pleiotropic activities, a critical limitation is that the effective concentrations employed in many cell-based assays (typically ranging from 10 to 100 μM) frequently surpass the plasma or tissue levels attainable following topical or oral administration. For topical delivery, the actual concentration reaching viable skin layers is governed by formulation characteristics, skin permeation efficiency, and the physicochemical properties of PIC itself (logP~2.5, aqueous solubility < 0.1 mg/mL). Thus, the direct extrapolation of in vitro potency to in vivo efficacy requires caution. A critical consideration when interpreting the preclinical evidence is the variability across experimental models. Immortalized cell lines (e.g., HaCaT keratinocytes, B16F10 melanoma cells) offer high throughput and mechanistic tractability but may not fully recapitulate the differentiated phenotype or barrier function of primary human cells. Ex vivo human skin explants offer a more physiologically relevant architecture and cellular heterogeneity but are constrained by limited availability and donor-to-donor variability. In vivo animal models, while enabling systemic assessment, may not accurately reflect human skin pharmacokinetics or immune responses. Additionally, the concentrations of PIC used in in vitro studies typically range from 1 to 100 μM, with the lower end of this range (1–10 μM) being more likely to approximate concentrations achievable following topical application of optimized formulations. Future studies should systematically characterize dose–response relationships, including potential hormetic effects: low doses of polyphenols may activate adaptive stress responses, while higher doses can become cytotoxic.

Beyond conventional two-dimensional cell culture models, emerging evidence indicates that PIC exhibits dermal bioactivity in more physiologically relevant human skin models. For instance, Yoshihara et al. (2024) demonstrated that PIC treatment in human dermal fibroblasts significantly upregulated hyaluronic acid synthase 2 (HAS2) expression and increased hyaluronic acid content via SIRT1-mediated mechanism [42]. Additionally, PIC-rich *Passiflora edulis* seed extracts have been shown to accelerate wound closure in keratinocyte monolayers and reduce UVB-induced morphological alterations in organotypic skin models [40,43]. These findings concertedly enhance the translational significance of PIC by demonstrating its efficacy in complex, multicellular systems that more closely mimic native human skin architecture and function.

### 3.3. Targeting Hyperpigmentation and Acne

PIC demonstrates potent anti-melanogenic efficacy in b16F10 melanoma cells. It reduces melanin content more effectively than resveratrol at equivalent concentrations and exerts its depigmenting effects by suppressing microphthalmia-associated transcription factor (MITF) and tyrosinase expression and activity; similar MITF/tyrosinase-targeting mechanisms have also been reported in other pigmented melanoma models and in PIC-rich PFS extracts [9,16,44]. Recent evidence extends this mechanism, showing that PIC-rich fractions can attenuate UVB-induced F-actin polymerization in melanocytes, potentially limiting dendrite formation and melanosome transfer [45]. For acne management, an ethanolic seed extract from purple passion fruit (*Passiflora edulis* sims var. edulis) exhibited notable bacteriostatic activity against *C. acnes* in agar diffusion assays, with inhibition zones at 5% extract comparable to clindamycin; this activity has been associated with the high pic content of the seeds [46]. Given the structural features of polyphenols like PIC, they may also function as photosensitizers in antimicrobial photodynamic therapy—a promising strategy against resistant skin pathogens [47,48].

**Table 1:** Summary of key preclinical studies on the dermatological effects of piceatannol.

Biological Activity	Model System	Key Findings	Relevance to Cosmeceutical Claims	Representative Refs.
Antioxidant & Anti-inflammatory	Human keratinocytes (HaCaT), murine macrophages (RAW 264.7)	PIC scavenges ROS and suppresses LPS-induced pro-inflammatory cytokines (TNF-α, IL-6) via inhibition of NF-κB and MAPK signaling pathways.	Soothing, redness reduction, anti-pollution, barrier support	[33,34,36,37,38,41]
Anti-photoaging	Human dermal fibroblasts (HDFs), UVB-irradiated HaCaT cells	PIC inhibits UVB-induced MMP-1/3 expression and collagen degradation; enhances type I procollagen synthesis via SIRT1 activation. Superior to resveratrol in some assays.	Anti-wrinkle, firming, collagen-boosting, photoprotection booster	[39,40,41]
Hydration & Extracellular Matrix Support	HDFs, 3D human skin equivalents	PIC upregulates hyaluronic acid synthase 2 (HAS2), increasing HA production; stimulates collagen and elastin synthesis.	Deep hydration, plumping, and skin elasticity improvement	[42]
Anti-melanogenic (Brightening)	B16F10 murine melanoma cells, α-MSH-stimulated human melanocytes	PIC reduces melanin synthesis by downregulating MITF and tyrosinase expression, without cytotoxicity at effective doses.	Brightening, even skin tone, and hyperpigmentation correction	[9,16,44,49]
Antibacterial (Acne-targeted)	*Cutibacterium acnes* cultures	PIC exhibits direct bacteriostatic activity against *C. acnes*, with MIC values in the low μg/mL range.	Acne prevention, purifying, microbiome-balancing	[43,46]

## 4. Bridging Preclinical Findings and Clinical Application: The Defining Gap

While preclinical data are compelling, translational evidence from animal and human studies, though promising, reveals a critical disconnect necessary for dermocosmetic validation.

### 4.1. Animal Studies: Systemic Benefits with Skin Relevance

In vivo studies primarily elucidate PIC’s systemic, sirtuin-mediated effects. In high-fat diet-fed mouse models of metabolic disturbance, oral PIC administration (in the 10–40 mg/kg/day range) upregulates hepatic SIRT1, SIRT3, and SIRT6 expression, activates AMPK-related pathways, and attenuates systemic inflammation and adiposity-related disturbances [50]. These improvements in metabolic-inflammation tone, a key contributor to systemic “inflammaging” provide a plausible physiological basis for indirect skin benefits, such as the improved hydration reported in human trials. However, these studies lack direct measurement of cutaneous endpoints (e.g., skin biomechanics, histology), leaving the skin-specific efficacy inferred rather than conclusively proven.

### 4.2. Lack of Human Evidence and Standardized Methodologies (Table 2)

An 8-week randomized, placebo-controlled trial in women with dry skin found that oral supplementation with a PIC-rich PFS extract (5 mg piceatannol/day) significantly increased stratum corneum hydration, but the underlying molecular mechanisms and skin structure were not assessed [51]. An open-label pilot study using a 10% purple PFS extract cream in patients with acne vulgaris reported marked reductions in inflammatory and non-inflammatory lesions, yet the absence of a control group and the unquantified PIC content preclude definitive conclusions [52]. In a separate open-label trial, a 3% purple PFS extract cream applied to photoaged facial skin improved clinical aging scores, although the lack of a placebo control and mechanistic or PIC content analyses similarly limit interpretation [53].

Notably, a recent randomized, double-blind, placebo-controlled trial in 281 healthy adults demonstrated that oral administration of PIC (100 mg/day) derived from PFS extract significantly upregulated sirtuin mRNA expression in peripheral blood, with particularly pronounced effects in postmenopausal women [54]. This provides robust pharmacodynamic evidence of systemic sirtuin activation by PIC in humans; however, skin-specific outcomes or tissue-level biomarker assessments were not included [53,54,55,56].

**Table 2:** Critical Appraisal of Existing Human Studies on Piceatannol for Skin Health.

Study Design	Intervention	Primary Dermatological Finding	Critical Limitation
RCT (Oral)	Passion fruit seed extract providing 5 mg PIC/day for 8 weeks	Significantly improved skin hydration (corneometer measurement)	Lack of correlation with skin-level biomarkers (e.g., HAS2, AQP3); unknown contribution of other extract components [57]
Open-label (Topical)	3% passion fruit seed extract cream applied twice daily for 8 weeks	Reduced inflammatory acne lesions in patients with mild-to-moderate acne	No vehicle control; extract was not chemically standardized for PIC content or other actives [57]
RCT (Oral)	Passion fruit seed extract providing 100 mg piceatannol/day for 4 weeks	Increased SIRT1 mRNA expression in whole blood	No direct skin-related clinical or molecular endpoints assessed; relevance to dermal SIRT1 activity remains speculative [51,52,53]

The existing human studies on PIC for skin health suffer from several methodological limitations that constrain their interpretability and reproducibility. First, sample sizes are generally small (typically *n* < 50 per arm), increasing the risk of type II errors and limiting generalizability. Second, the majority of topical studies employ open-label designs lacking vehicle control, rendering the observed effects susceptible to placebo response and investigator bias. Third, none of the studies to date have used chemically standardized extracts with defined PIC content as a critical quality attribute, making it difficult to attribute clinical outcomes specifically to PIC rather than to co-extracted phytochemicals. Fourth, the absence of mechanistic biomarkers (e.g., SIRT1 activity, MMP-1 expression, or HAS2 levels) in skin tissue samples precludes the establishment of a pharmacodynamic link between PIC exposure and clinical response [58,59,60].

### 4.3. The Translational Gap: A Summary

Thus, a definitive translational gap exists, characterized by: (i) No mechanistic link in human skin between clinical improvement and modulation of targets like SIRT1 or MMP-1 [42,54,55]; (ii) No data on standardized, topically delivered PIC from optimized formulations [20,21,23,57]; and (iii) No OECD-compliant dermal safety profile [56,57]. Consequently, PIC resides in a “translational valley” between a promising nutraceutical and a validated cosmeceutical active ingredient. Advanced delivery systems—particularly polymeric nanoparticles derived from natural polymers—offer a rational strategy to enhance skin retention and bioactivity of such polyphenols, yet their clinical implementation remains limited despite strong preclinical promise [22]. Notably, a structurally related stilbenoid, resveratrol, has recently been reported to exhibit anti-aging and skin-brightening effects in preclinical studies when formulated as nanoliposomes, with enhanced transdermal delivery demonstrated using ex vivo skin models [58,59].

## 5. Towards a Circular Dermocosmetic Ingredient: A Translational Framework

**Figure 4:**
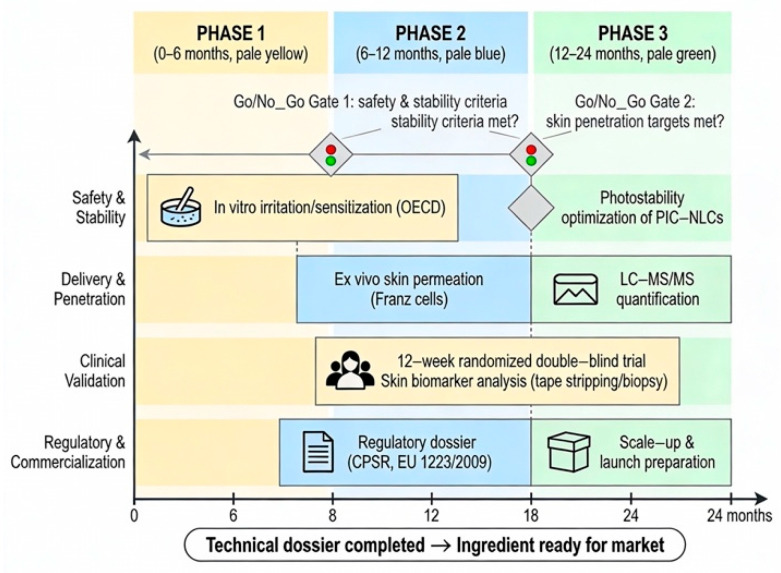
Proposed 24-month phase-gate translational pipeline for piceatannol derived from passion fruit seed waste. The pipeline consists of three phases: Phase 1 (Months 0–6) focuses on safety profiling and photostabilization using nanostructured lipid carriers (NLCs); Phase 2 (Months 6–12) involves ex vivo skin permeation and deposition studies; Phase 3 (Months 12–24) encompasses a randomized, double-blind, vehicle-controlled clinical trial with correlative skin biomarker analysis. Key deliverables and decision gates are indicated.

The promising yet fragmented dossier on PIC points to an integrated translational strategy. This section synthesizes a testable development framework, conceived within circular bioeconomy principles, to systematically convert PFS waste into a standardized, efficacious, and commercially viable cosmeceutical bioactive [2,42,60,61]. The framework is predicated on two non-negotiable foundations and proceeds via a phase-gate experimental pipeline (Figure 4).

### 5.1. Foundational Imperative I: Establishing Phytochemical Standardization

Analytical data from optimized extraction studies indicate that PIC-enriched PFS extracts with PIC contents in the mg/g range can be achieved using techniques such as pressurized liquid extraction (PLE) [62,63]. Based on this evidence, we propose a minimum PIC content of ≥0.3% (*w*/*w*). This threshold is derived from the reported PIC content in *Passiflora edulis* seeds obtained via optimized extraction techniques such as pressurized liquid extraction (PLE), which typically yields PIC concentrations in the range of 0.3–3.68% (*w*/*w*) depending on cultivar, extraction solvent, and processing conditions [4,5,62]. The proposed lower limit of 0.3% represents a practically achievable benchmark that balances analytical feasibility, batch-to-batch consistency, and the requirement for sufficient bioactivity in dermal applications, while remaining technically and economically viable for industrial-scale production. This threshold, alongside compulsory CQAs covering identity confirmation (e.g., HPLC-HRMS profiling) and limits for key contaminants (pesticides, heavy metals, residual solvents) [59,63,64,65] would ensure batch-to-batch consistency and scientific reproducibility in future research and product development.

### 5.2. Foundational Imperative II: Implementing a Cascading Biorefinery for Sustainable Sourcing

**Figure 5:**
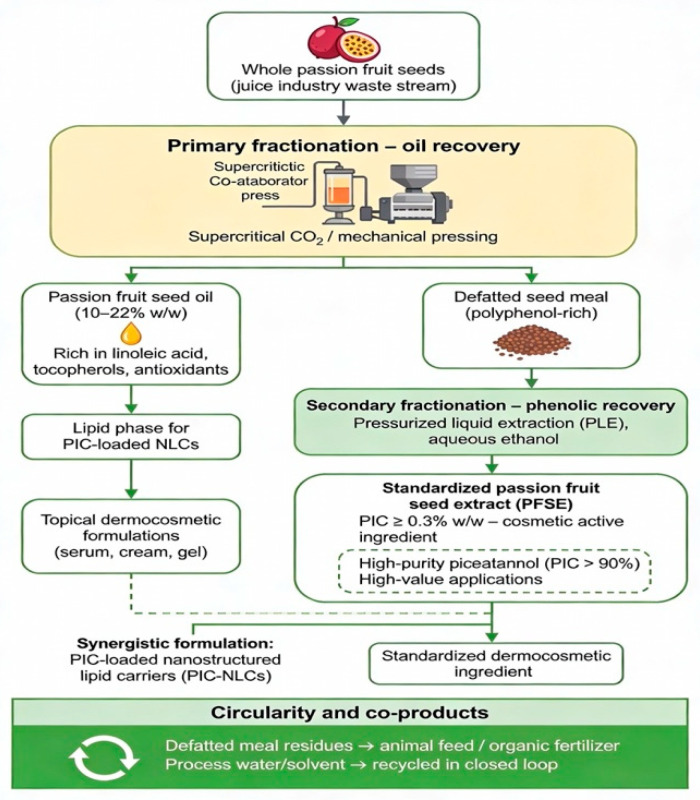
Cascading biorefinery model for passion fruit seeds. The process begins with whole seeds, which undergo primary fractionation for oil recovery, yielding passion fruit seed oil (emollient and bioactive lipid phase). The defatted seed meal is then subjected to secondary fractionation via pressurized liquid extraction (PLE) to recover phenolic compounds, including piceatannol. Residual fiber may be utilized as animal feed or filler. This integrated approach maximizes resource efficiency and aligns with circular bioeconomy principles.

Achieving economic and environmental sustainability requires a holistic valorization strategy that extends beyond the single-compound isolation. A cascading biorefinery model (Figure 5) maximizes resource efficiency and creates intrinsic product synergy.

**Primary Fractionation (Oil Recovery):** Initial oil recovery from whole PFS—via established methods such as mechanical pressing or solvent extraction—can yield a substantial lipid fraction, with reported oil contents typically ranging between 10% and 22% (*w*/*w*) on a dry weight basis [1,64]. The high linoleic acid content, together with naturally occurring tocopherols, imparts both emollient and antioxidant properties to the oil. Critically, it is not merely a by-product; the oil can serve as a sustainable, bioactive lipid phase for the formulation of PIC-loaded nanocarriers (e.g., nanostructured lipid carriers) [20], thereby linking upstream biomass valorization to downstream delivery system design.

**Secondary Fractionation (Phenolic Recovery):** The defatted PFS, now enriched in polyphenols, undergoes optimized pressurized liquid extraction (PLE) with green solvents (e.g., aqueous ethanol) to produce a standardized PFS extract. Analytical studies indicate that PIC-enriched PFS extracts with contents in the mg/g range can be achieved [62]; further purification steps may yield high-purity PIC (>90%) for specialized applications [66]. This sequential approach is conceptually validated by biorefinery studies on *Passiflora* seeds, where co-utilization of oil and antioxidant-rich defatted extracts enhanced overall product stability and functionality [67]. Beyond seed valorization, the peel of purple passion fruit—rich in anthocyanins, regulated by *PeMYB114* [68]—represents another underutilized stream for natural colorants or antioxidant extracts, further supporting a whole-fruit biorefinery paradigm. Notably, passion fruit processing residues are also being explored in the food sector for upcycled flour production, where seed- and pulp-containing by-products are milled into nutrient-dense ingredients that enhance fiber and phytochemical content in baked goods [69].

From an economic feasibility perspective, the cascading biorefinery model improves overall profitability by generating multiple revenue streams: PFS oil (emollient applications), PIC-enriched extracts (cosmeceutical active), and residual fiber (potential filler or animal feed). Preliminary techno-economic assessments of similar fruit seed valorization processes suggest that integrated biorefinery configurations can achieve positive net present value (NPV) when total biomass utilization exceeds 80% and when value-added fractions command premium pricing in the cosmeceutical market. Industrial scalability is supported by the availability of commercial-scale extraction equipment (e.g., screw presses for oil recovery, pressurized liquid extractors for phenolic fractionation) and existing supply chains for passion fruit processing waste. Future life-cycle assessment (LCA) studies will be necessary to quantify the environmental footprint of this valorization pathway, with key impact categories including global warming potential (GWP), water consumption, and land use, compared to conventional disposal methods such as landfilling or incineration.

This “cradle-to-cosmeceutical” model aligns with circular bioeconomy principles, enabling near-total biomass utilization, minimizing waste, and economically justifying the upstream processing of an agricultural residue [2].

### 5.3. A Phase-Gate Translational Pipeline: From Standardized Extract to Clinical Validation

With a standardized PFS extract established, the focus shifts to building the robust technical dossier required to navigate regulatory approval and ensure market entry. A 24-month phase-gate pipeline is proposed as an illustrative and hypothesis-driven framework rather than a prescriptive guideline.

**Phase 1: Safety and Fundamental Stability (Months 0–6)**

**Objective:** Establish a preliminary safety margin and overcome inherent physicochemical instability.
**Key activities:**

- **Safety profiling.** Conduct in vitro skin irritation and sensitization assays in line with OECD guidelines (e.g., reconstructed human epidermis models for irritation and ARE-Nrf2 luciferase assays for sensitization) on the standardized PFS extract compared to the PIC reference standard, addressing the current lack of dermal safety data for this stilbene [69]. Beyond initial in vitro irritation and sensitization assays, a comprehensive dermal safety dossier should include phototoxicity testing (e.g., 3T3 NRU phototoxicity assay per OECD 432), given PIC’s potential to absorb UV radiation and its intended use in topical formulations. Repeated-dose toxicity studies in reconstructed human epidermis models can provide insights into cumulative irritation potential and barrier disruption following prolonged exposure. To leverage existing toxicological knowledge, read-across from the structurally related, more extensively characterized polyphenol resveratrol is scientifically justified, as both share a stilbene backbone and are metabolized via similar phase-II pathways. Available data indicate that resveratrol exhibits a favorable dermal safety profile with no evidence of genotoxicity or significant phototoxicity at concentrations up to 10% in topical formulations; however, confirmation for PIC and PIC-rich extracts is required due to the additional catechol moiety, which may alter redox activity and toxicokinetic properties.
- **Photostabilization.** Formulate PIC within PFS oil-based nanostructured lipid carriers (NLCs) and test the hypothesis that such encapsulation significantly reduces UVA/UVB-induced photodegradation compared to an unformulated control using established photostability protocols adapted from ICH Q1B and prior NLC photostability work. While systemic delivery differs from topical application, the principle that nanoencapsulation can profoundly enhance the stability and bioactivity of PIC is further supported by recent advances in nanomedicine, including a red blood cell membrane-coated biomimetic nanoparticle that achieved sustained release of PIC under hypoxic conditions [70]. This reinforces the rationale for exploring lipid-based nanocarriers in dermal delivery. Moreover, the compatibility of antioxidant-loaded NLCs with nanosized UV filters in stable, non-irritating topical gels has been recently demonstrated, supporting the feasibility of integrating PIC-NLCs into multifunctional photoprotective formulations [71].

**Phase 2: Topical Delivery and Bioperformance (Months 6–12)**

**Objective:** Quantify and optimize cutaneous bioavailability.
**Key activity:**

- Perform ex vivo permeation and deposition studies using human skin mounted on Franz-type diffusion cells, comparing PIC-loaded NLCs with a conventional oil-in-water emulsion. Since the skin permeability of phenolic compounds is highly dependent on molecular characteristics, it can be preliminarily assessed using in silico Log Kp modelling [72]. The working hypothesis is that NLCs will substantially enhance PIC deposition in the stratum corneum and viable epidermis compared to a conventional formulation, consistent with improvements reported for other actives in lipid nanocarriers, with LC-MS/MS used for quantitative analysis [20,21,71].

**Phase 3: Biomarker-Correlated Clinical Proof-of-Concept (Months 12–24)**

**Objective:** Obtain preliminary efficacy evidence with mechanistic insight.
**Key activity:**

- Execute a randomized, double-blind, vehicle-controlled clinical trial of a PIC-NLC formulation at a concentration and duration aligned with prior cosmeceutical studies on similar bioactives [55,73]. We hypothesize that the intervention will produce measurable and statistically significant gains across objective endpoints (e.g., skin hydration, elasticity, pigmentation) while, critically, incorporating correlative skin biomarker analysis—such as minimally invasive biopsies or tape-stripping to assess changes in genes/proteins such as SIRT1, HAS2, and MMP-1, which have been implicated in PIC’s dermal actions in vitro and in the biology of skin aging [39,42,50,55].

### 5.4. Regulatory Integration and Commercial Pathway

Proactive regulatory strategy is integral to this framework. As PIC is not currently listed as a standalone cosmeceutical ingredient in major inventories such as the EU Cosing Database, any PIC-containing dermocosmetic will require a complete safety assessment dossier, for example, a Cosmetic Product Safety Report (CPSR) under EU Regulation (Ec) No 1223/2009 [74,75]. Given the EU ban on animal testing for cosmetics, such dossiers must rely on new approach methodologies (NAMS), including read-across from structurally related compounds (e.g., resveratrol), in silico toxicology, physiologically based kinetic (PBK) modeling, and integrated in vitro assays—as recently demonstrated in a next-generation risk assessment case study for resveratrol [76]. The data generated from phases 1–3 would constitute the core of this submission by addressing ingredient identity, purity, safety, exposure, and claim substantiation requirements. independent, third-party verification of all CQAs is essential to ensure regulatory compliance, mitigate the well-documented risk of botanical ingredient adulteration, and build brand integrity [77].

### 5.5. Synthesis: A Blueprint for Action

This review has articulated a clear vision and a structured pathway. The journey for PFS-Derived PIC is now delineated. The proposed framework addresses the central challenge of natural product development: balancing source variability and sustainable sourcing with the need for reproducible, evidence-based efficacy. By mandating upfront standardization, adopting an integrated biorefinery, and executing a targeted clinical pipeline with mechanistic depth, this framework provides a replicable blueprint. Implementation through cross-sectoral consortia could establish PIC as a pioneering paradigm for the next generation of circular, evidence-based dermocosmetic bioactives [11].

### 5.6. Technology Readiness Level and Implementation Bottlenecks

The proposed translational pipeline can be mapped onto the Technology Readiness Level (TRL) scale commonly used to assess innovation maturity. Currently, PIC from *Passiflora edulis* seeds resides at TRL 2–3, with its basic principles established (TRL 2) and preliminary proof-of-concept demonstrated in in vitro and ex vivo models (TRL 3). The framework outlined herein aims to advance PIC to TRL 5–6, where validated prototypes are tested in relevant environments (clinical trials). Critical bottlenecks that must be addressed include: (i) regulatory hurdles, particularly the need for a complete Cosmetic Product Safety Report under EU Regulation (EC) No 1223/2009 in the absence of a pre-existing monographic entry; (ii) scalability challenges associated with the cascading biorefinery model, including capital expenditure for extraction and purification equipment and the need for consistent biomass supply chains; and (iii) standardization of critical quality attributes across different *Passiflora edulis* cultivars and geographical sources. Proactive engagement with regulatory bodies, investment in pilot-scale processing infrastructure, and the establishment of industry-wide consensus standards will be essential to overcome these barriers.

### 5.7. Broader Applicability to Other Agricultural Waste Valorization

The translational framework proposed here—centered on compound-specific standardization, cascading biorefinery integration, and a phase-gate clinical development pipeline—extends beyond *Passiflora edulis* seeds, offering a generalized model for harnessing and valorizing other bioactive-rich agricultural byproducts. For instance, similar approaches can be applied to grape pomace (rich in resveratrol and proanthocyanidins), olive mill waste (rich in hydroxytyrosol), or mango seed kernels (rich in gallic acid and mangiferin). By adopting this paradigm, the cosmeceutical and nutraceutical industries could systematically transform underutilized byproducts into standardized, evidence-based ingredients, thereby advancing both circular bioeconomy goals and product innovation. The structured, stepwise nature of the framework—mandating upfront standardization, integrated biorefinery design, and biomarker-correlated clinical validation—ensures that each stage of development is both scientifically rigorous and commercially informed, facilitating translation across diverse waste streams and regulatory contexts.

## 6. Conclusions

Passion fruit seed waste represents a sustainable and underutilized source of PIC, a multi-target stilbenoid with compelling preclinical evidence for modulating key pathways in skin aging, hyperpigmentation, and acne. Nevertheless, its translation into a reliable skin-targeted bioactive has been impeded by a lack of standardized extracts, insufficient safety and delivery data, and a paucity of clinically validated efficacy—a translational gap this review systematically identifies and seeks to address.

To bridge this gap, we propose a circular, bioeconomy-aligned roadmap that mandates establishing a ≥0.3% (*w*/*w*) PIC threshold as a critical quality attribute for extract standardization, ensuring batch-to-batch consistency and reproducible bioactivity. Furthermore, we advocate an integrated, cascading biorefinery model that co-produces PFS oil and phenolic fractions, maximizing resource efficiency and enabling intrinsic synergies for formulation development. Finally, we outline a staged, hypothesis-driven translational pipeline encompassing safety assessment, photostabilization via nanodelivery systems, ex vivo skin penetration studies, and biomarker-correlated clinical trials.

Successful execution of this roadmap requires interdisciplinary collaboration spanning agronomy, phytochemistry, cosmeceutical formulation science, dermatology, and clinical research. Its implementation promises not only to position PFS-derived PIC as a benchmark for evidence-based, sustainable cosmeceuticals but also to establish a replicable model for the valorization of other bioactive-rich agricultural waste streams, thereby contributing to a more resource-efficient and scientifically grounded skincare industry.

## Data Availability

No new data were created or analyzed in this study.

## Abbreviations

The following abbreviations are used in this manuscript:

**Abbreviation**	**Full Form**
PIC	Piceatannol
PFS	Passion fruit seed
CQAs	Critical quality attributes
NLCs	Nanostructured lipid carriers
SIRT1	Sirtuin 1
HAS2	Hyaluronan synthase 2
MMP-1	Matrix metalloproteinase-1
MITF	Microphthalmia-associated transcription factor
*C. acnes*	*Cutibacterium acnes*
ROS	Reactive oxygen species
NF-κB	Nuclear factor kappa-light-chain-enhancer of activated B cells
MAPK	Mitogen-activated protein kinase
OECD	Organisation for Economic Co-operation and Development
